# Waning Immunity Is Associated with Periodic Large Outbreaks of Mumps: A Mathematical Modeling Study of Scottish Data

**DOI:** 10.3389/fphys.2017.00233

**Published:** 2017-04-25

**Authors:** Dalila Hamami, Ross Cameron, Kevin G. Pollock, Carron Shankland

**Affiliations:** ^1^Department of Computing Science, University of Oran1 Ahmed BenBellaOran, Algeria; ^2^Health Protection ScotlandGlasgow, UK; ^3^Department of Computing Science and Mathematics, University of StirlingStirling, UK

**Keywords:** mumps, vaccination, waning immunity, mathematical and computational modeling and simulation, Bio-PEPA

## Abstract

Vaccination programs for childhood diseases, such as measles, mumps and rubella have greatly contributed to decreasing the incidence and impact of those diseases. Nonetheless, despite long vaccination programmes across the world, mumps has not yet been eradicated in those countries: indeed, large outbreaks continue. For example, in Scotland large outbreaks occurred in 2004, 2005, and 2015, despite introducing the MMR (Measles-Mumps-Rubella) vaccine more than 20 years ago. There are indications that this vaccine-preventable disease is re-emerging in highly vaccinated populations. Here we investigate whether the resurgence of mumps is due to waning immunity, and further, could a booster dose be the solution to eradicate mumps or would it just extend the period of waning immunity? Using mathematical modeling we enhance a seasonally-structured disease model with four scenarios: no vaccination, vaccinated individuals protected for life, vaccinated individuals at risk of waning immunity, and introduction of measures to increase immunity (a third dose, or a better vaccine). The model is parameterised from observed clinical data in Scotland 2004–2015 and the literature. The results of the four scenarios are compared with observed clinical data 2004–2016. While the force of infection is relatively sensitive to the duration of immunity and the number of boosters undertaken, we conclude that periodic large outbreaks of mumps will be sustained for all except the second scenario. This suggests that the current protocol of two vaccinations is optimal in the sense that while there are periodic large outbreaks, the severity of cases in vaccinated individuals is less than in unvaccinated individuals, and the size of the outbreaks does not decrease sufficiently with a third booster to make economic sense. This recommendation relies on continuous efforts to maintain high levels of vaccination uptake.

## Introduction

To prevent, control and eradicate childhood diseases, vaccination programs have been adopted throughout the world. For example the trivalent measles-mumps-rubella vaccine (MMR) (Harling et al., 2005; Le Menach et al., 2014; Cordeiro et al., 2015) has been highly successful for both measles and rubella reduction in many countries. Despite near eradication of both measles and rubella (Isaacs and Menser, 1990; Glass and Grenfell, 2004; Ueda, 2016), elimination of mumps has not been achieved and could be considered to be re-emerging, despite initial early success in reducing mumps cases. In the last decade, many countries, such as Belgium (Abrams et al., 2014), Korea (Park, 2015), the Netherlands (Snijders et al., 2012), and the US (Dayan et al., 2008) have reported a dramatic increase in the incidence of mumps. In Scotland, 2004/2005 saw a sudden high resurgence in mumps with approximately 4500 cases, 8 years after the second dose of MMR was included in the vaccination program (which was predicted to substantially reduce mumps outbreaks Anderson and May, 1992). One hypothesis is that the resurgence was related to declining vaccine coverage (Nardone et al., 2003; van Boven et al., 2013), in particular, a widespread scare related to autism which led to some parents refusing to vaccinate their children. This can be easily debunked: the herd immunity threshold is estimated at 75–86% (Donaghy et al., 2006) and mumps vaccination levels have stayed above that level (e.g., in Scotland, ranging from 87 to 94% pre-2004). In addition Donaghy et al. (2006) argues that those infected during the 2004/2005 epidemics are characterized by low uptake of a single dose of MMR (catch-up campaign) and being of school age at time when the mumps virus had greatly reduced circulation in that group, delaying infection. The study undertaken by DeStefano et al. (2013) analyzing the number of antigens in both children with and without autism, shows that there is no association between receiving vaccine and developing autism.

A second hypothesis is to link vaccination status and age, e.g., proposing that outbreaks continue in the older population but die out in the increasingly vaccinated population. However, while age structure has shown to be informative in many models of traditionally childhood diseases (Andreasen, 1993; Ferguson et al., 1996; Hethcote, 2000; Brisson et al., 2010), current studies suggest that age is not the key determinant in mumps. Snijders et al. (2012) do not find any significant interaction between these two features. In addition, several studies of different outbreaks occurring at different times and locations in the US and Canada (Centers for Disease Control and Prevention, 2009) indicate that there is no evidence that age is the main factor leading to mumps spread. For instance, the outbreaks occurring in New York (Sulivan, Brooklyn, Rockland county and Orange county), New Jersey and Canada show variable average of infected age groups (Sullivan: 12 years, Brooklyn: 14 years, Rockland county: 12 years, Orange county: 18 years, New Jersey: 19.5 years and Canada: 27.5). However, it was confirmed that all cited cases were related to religious events or camping in Sullivan, with the majority fully vaccinated. It was also reported that the series of outbreaks were due to one fully vaccinated child aged 11 years who had been infected during his travel to UK. Snijders et al. (2012) analyzed a group of infected whose ages ranged in 3–13 years. The authors find out that no significant difference between the attack rate of the group aged 10–13 years and 3–5 years. Considering Scotland specifically, Donaghy et al. (2006) argued that the shift of ages observed in the epidemic in Scotland suggests that the propagation of mumps is becoming more widespread and diverse as the targeted population becomes more dynamic and mobile.

Having rejected the first two hypotheses, the arguments used lead to the third and more plausible hypothesis: MMR vaccine efficacy against mumps reduces over time (van Boven et al., 2013). In 2015 67% of those infected in Scotland were fully vaccinated individuals (1 and 2 doses confounded). Moreover, most primary cases occurred in adolescent and young adults, in contrast to the pre-vaccine era where outbreaks were among children of primary school age. Similar patterns can be found for Belgium in 2012 (Abrams et al., 2014) and in the US in 2006 (Dayan et al., 2008). Serological studies (Heffernan and Keeling, 2009; Park, 2015) show that susceptibility level increases (immunity wanes) as time from vaccination increases; however, the antibody threshold defining the protective level is not well specified for mumps (LeBaron et al., 2009). Even using two doses of the MMR vaccine, existing analyses (Cameron and Smith-Palmer, 2015; Park, 2015) stress that some of the population will remain at risk of disease unless additional control strategies are adopted.

We investigate the hypothesis of waning immunity using mathematically-based computational modeling. The basic model is a seasonal compartmental SEIR model (Anderson et al., 1984, 1987; Keeling and Grenfell, 2002), to which vaccination and immunity is added. We first show that the model produces comparable results to observed mumps data in Scotland^1^, matching endemic levels of mumps with occasional larger epidemics, as in 2005 and 2015. Having established the accuracy of the model with historical data, we use it predictively to better understand the relationship between immunity and transmission, to illuminate long-term patterns of resurgent outbreaks, and to determine whether these can be controlled by extending immunity duration (e.g., by using another booster). While modeling has been previously used to investigate mumps and vaccination (Anderson et al., 1987; Abrams et al., 2014; Edmunds et al., 2000), the novelty of our approach lies in consideration of waning immunity and associated optimal control strategies. Our model shows clearly that waning immunity is a driver for a long period of oscillating outbreaks. Moreover, by working with epidemiologists to use mathematics to understand the observed clinical data, we illustrate the power of mathematics to inform public health policy through multi-disciplinary collaboration.

## Mumps epidemiology in Scotland

**Figure 1:**
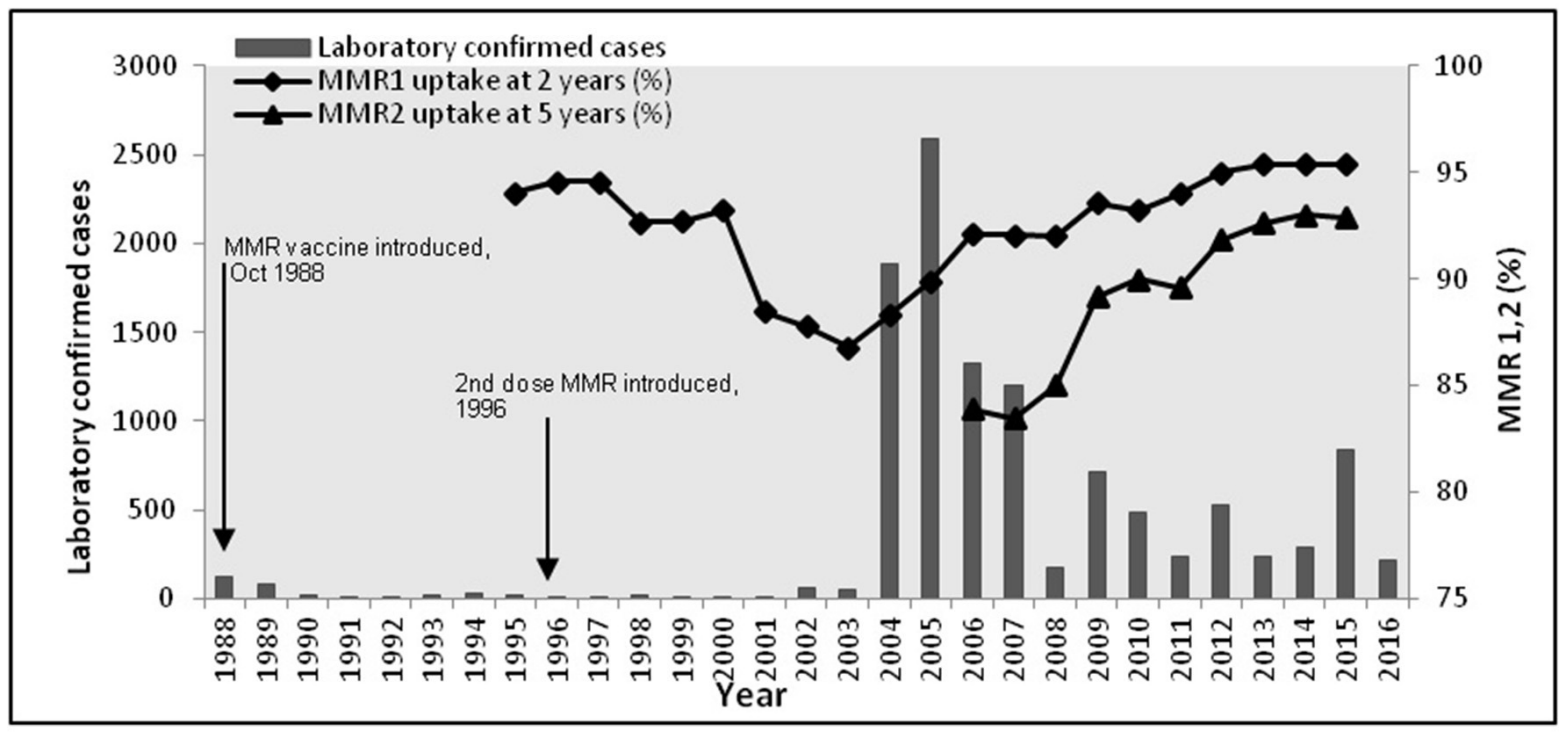
**Confirmed mumps cases, Scotland 1988-2016 and MMR vaccine coverage**.

During the period 1988–2015, Health Protection Scotland (HPS), the national surveillance center for Scotland, reported 10943 mumps cases. 10486 of these cases were between 2004 and 2015. Vaccination was introduced in 1988, with a second dose introduced in 1996. Figure 1 shows the epidemic curve of mumps, and the vaccination uptake curves for both vaccines (MMR1 and MMR2). Observe the initial success of the vaccine (1988–2003) contrasted with a long potential cycle from 2004 to 2015, possible with sub-cycles (2005–2009, 2009–2012, 2012–2015). The 2004/2005 outbreak was related only partly to the decrease in vaccination coverage shown in Figure 1 (Donaghy et al., 2006). The majority of cases (94%) were born before 1990 (aged 15+ years), with only a few of them receiving only one dose of MMR (around 1%) or none at all. Similarly for the outbreaks in 2009 and 2012. In 2015 the highest incidence of mumps (63%) was related to the group born 1991–2000 (aged 15–24 years). Cameron and Smith-Palmer (2015) argue that the 2015 outbreak was the first where the majority of cases were fully vaccinated. Transmission is a complex feature to model as it can be influenced by many factors (vaccination history, current immunity status, age, opportunity for social mixing, geography, and so on). Moreover, some of these factors are confounded (e.g., age and vaccination history). We propose in this model that vaccination history is used as a proxy for these combined effects. Therefore, the main question arising is: why are vaccinated individuals being infected? Here we focus on the long curve (2005–2015) relating to the long inter-epidemic period. We explore these features within the model presented in Methods, using the Bio-PEPA plugin tool (Ciocchetta and Hillston, 2009) and deterministic simulation to provide time series prediction of the number of infected individuals. The model is parameterised and validated on data up to 2015, and then to further validate its predictive performance it is shown to match 2016 data provided by HPS. The advantages for using the Bio-PEPA formalism (a mathematically-defined computational modeling approach called process algebra) have been fully argued in many works (Ciocchetta and Hillston, 2009; Benkirane et al., 2012; Hamami and Atmani, 2013). Here, the advantages are: formal structuring of interactions between components, a compositional approach to building the epidemiological model, and a range of analysis techniques to support the modeler in understanding the system. The underlying semantics of Bio-PEPA is a continuous time Markov chain.

## Methods

### Model structure, epidemiological assumptions, and parameter estimates

**Figure 2:**
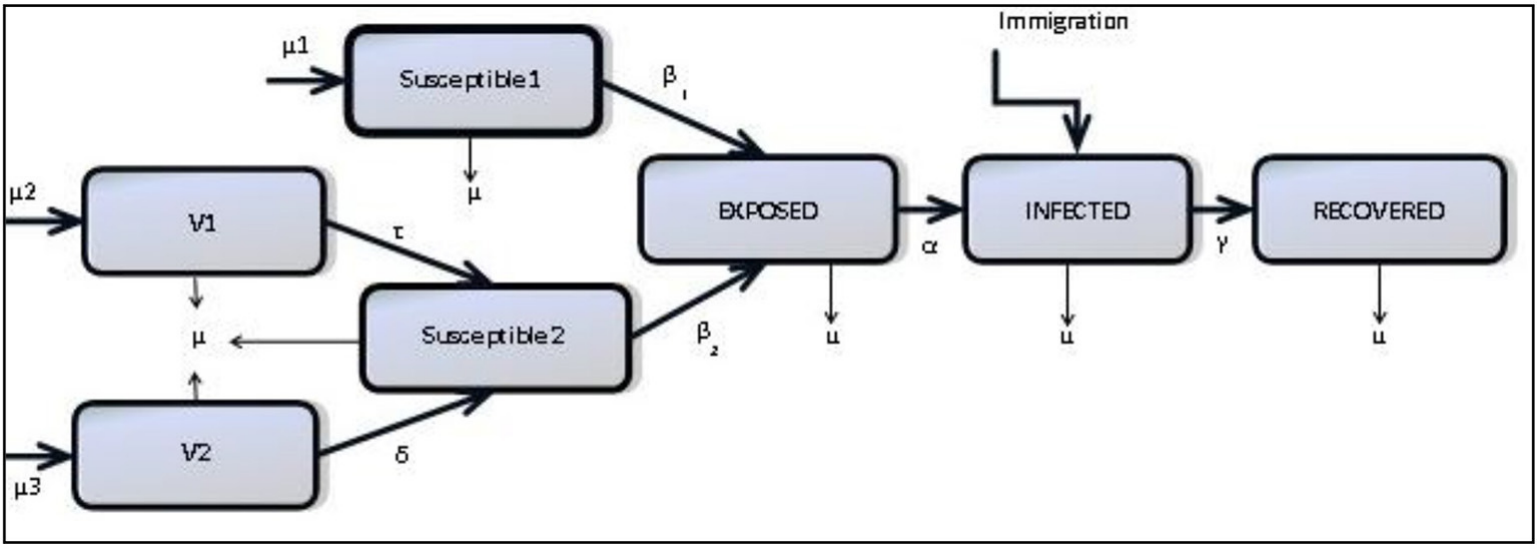
**Mumps structure**.

We consider a compartmental structure for a model of mumps formulated as an extended SEIR (Anderson and May, 1992) model including seasonality and waning immunity: natively susceptible (S1), vaccinated individuals with MMR1 only (V1), vaccinated individuals with both MMR1 and MMR2 (V2), modified susceptible who are vaccinated individuals who have become susceptible (S2), exposed individuals (E), infected individuals (I) and recovered individuals who are regarded as immune for life (R) (Anderson and May, 1992; Greenhalgh and Sfikas, 2003). Figure 2 shows how these compartments interact.

Our goal is to provide as simple a model as is necessary to demonstrate the impact of waning immunity, therefore we have ignored features which others have chosen to include. For example, the models of Glass and Grenfell (2003) and Barbarossa and Röst (2015) include immunity levels and immune-boosting through vaccination and interactions with infected. Since we have no data on antibody levels as individuals interact we choose not to include this, choosing the simpler scenario which can be parameterised through observed data. Neither do we include age-structure, as mumps has ceased to be a mainly childhood disease. As shown in several works (Donaghy et al., 2006; Centers for Disease Control and Prevention, 2009; Brockhoff et al., 2010; Fanoy et al., 2011), the range of those infected with mumps has become more diverse due to a more mobile susceptible population. Therefore, rather than stratifying the population by age, we assume a more homogenously-mixed population, with routine vaccination, and transmission based on seasonality and immunity status.

This model is general and could be parameterised for any seasonal disease with up to two vaccinations. We use data from Health Protection Scotland (HPS) from 2004 to 2016^2^ and some parameters from the literature (Anderson and May, 1992; Keeling and Rohani, 2008). These are detailed in Table 1 Appendix 3 in Supplementary, with some explanatory text.

- Demographic estimation

Birth and death rate (μ) estimated from Scottish demographic data^3^.

- Immigration rate estimation (λ)

As the net migration to Scotland is insignificant (typically 15,000 per year), the model has been simplified by having neither mass emigration nor immigration of susceptible individuals. A small constant rate of immigration of infected individuals is required to prevent the disease dying out entirely. This is justified by the knowledge that there is immigration, and there are many populations in the world where mumps is more prevalent and the global population is more mobile, transmitting disease between countries. A small rate of immigration of infectious individuals is estimated as in Finkenstädt et al. (1998) and Benkirane et al. (2012).

- Vaccination rates estimation (μ_1_, μ_2_, μ_3_)

According to vaccination data^4^, our basic assumption is an average of 94% MMR1 vaccination coverage (1988–2016) for children aged 0 to 2 years and 90% MMR2 vaccination coverage (1996–2016) for children aged 3 to 5 years. According to past vaccination history (Morgan-Capner et al., 1988; Public Health England, 2013), we estimate the susceptible portion of the remaining unvaccinated population at 20%. Within that proportion of susceptible we consider 11% of those to be aged 10 years or over according to current demographics. It would be more realistic to consider a varying vaccination rate each year; however, we did not want this to confound the patterns obtained through simply waning immunity. We do investigate scenarios in which these average vaccination rates are varied across the simulation period, to show how this affects the pattern of outbreaks.

- Waning immunity estimation (τ, δ)

Our basic assumption is individuals vaccinated with MMR1 and MMR2 (resp. only MMR1) are temporarily protected and that immunity wanes toward susceptibility at constant rate δ (resp. τ). LeBaron et al. (2009) report low antibody levels 4–9 years after MMR1 only, and 7–12 years after MMR2 administration. We also investigate scenarios in which these rates are varied.

- Transmission rate estimation (β1, β2, β3)

In our model, the transmission rate depends on two features: seasonality (High, Low) and type of susceptible (native susceptible, modified susceptible) giving four rates: β1 (High season and native susceptible), β2 (high season and modified susceptible, β3 (low season and native susceptible), β4 (low season and modified susceptible). For seasonality, data report higher number of cases October to May, and fewer between June and September^5^. As most cases occurs in 17–24 year-olds this seasonality is further supported through an assumption that many of that group are likely to be in full-time education, and mixing more in semester-time than in the holiday. As the total number of infected at low season is small we assume β3 = β4. In addition, we assume β2 > β1 (transmission in modified susceptible is higher than in native susceptible). This follows from the model of Scherer and McLean (2002), and is supported by the report of Cameron^6^ that within 205 confirmed cases related to two health boards, 137 (67%) individuals were fully vaccinated. As transmission rate is based on the basic reproduction number R_0_ (see Table 1 Appendix 3 in Supplementary), a range of proposed values were collected from literature (Anderson et al., 1987; Anderson and May, 1992; van Boven et al., 2013), where R_0_ is ranged [4–11]. See Sensitivity Analysis for sensitivity analysis of the particular choices of these rates.

- Incubation rate α and recovery rate γ

Established empirical studies (Anderson et al., 1987; Anderson and May, 1992) estimate the incubation period between 12 and 25 days and the infectious period between 7 and 9 days (Anderson et al., 1987). For modeling convenience, we assume the same period of infection and incubation (Public Health England, 2013) for both natively susceptible and modified susceptible.

- Initial conditions

The initial mix of susceptible, vaccinated, exposed, infected and recovered is calculated for 1996 according to the above assumptions about population based on vaccination beginning in 1988. See Appendix 1 in Supplementary (model component).

The description of the model and parameters above can be summarized by seven ordinary differential equations:

$$\begin{array}{l} \;\frac{dS1}{dt}=\mu 1N-\frac{\beta \left(t\right)S1I}{N}-\;\mu \;S1\\ \;\frac{dV1}{dt}=\mu 2N-\tau V1-\;\mu \;V1\\ \frac{dV2}{dt}=\mu 3N-\;\delta V2-\;\mu \;V2\\ \;\frac{dS2}{dt}=\delta V2+\tau V1-\frac{\beta^{\prime }(t)S2I}{N}-\;\mu \;S2\\ \;\frac{dE}{dt}=\frac{\beta (t)S1I}{N}+\frac{\beta^{\prime }(t)S2I}{N}\;-\;\alpha E-\;\mu \;E\\ \;\frac{dI}{dt}=\alpha E-\;\gamma I-\;\mu I+\lambda \\ \;\frac{dR}{dt}=\gamma I-\;\mu R\\ \text{Where}:\\ \;\beta \left(t\right)\left(resp.\;\beta^{\prime }\left(t\right)\right)\\ \;=\{\begin{array}{l} \beta 1\;\left(resp.\;\beta 2\left(t\right)\right)\;if\;Time\;\in \left[October-May\right]\\ \beta 3\;if\;Time\;\in \left[June-September\right] \end{array} \end{array}$$

This model is coded in Bio-PEPA (see Appendix 1 in Supplementary). Analysis of the model is performed through deterministic simulation. Stochastic simulation was used to guide model development but does not provide additional information when identifying long term trends.

### Model scenarios

To capture the impact of vaccination efficacy and the effect of waning immunity on the population of Scotland for future projection of epidemics, the history of mumps epidemics (from pre-vaccine to post-vaccine era) are reproduced where four strategies are considered:

- *Scenario one*. No vaccination. This is equivalent to the pre-vaccine era and useful for model validation where the whole population is considered susceptible.
- *Scenario two*. Immunity does not wane: τ and δ are zero. This case reflects the introduction of a vaccination protocol to case one, where immunity is assumed to be for life. This is consistent with the period immediately following the introduction of vaccination.
- *Scenario three*. Immunity wanes in vaccinated individuals according to the assumptions above. This scenario reflects modern reality, where mumps is resurgent. Our model is extended to two separate but correlated models: the first model expresses unvaccinated individuals and the second model expresses vaccinated individuals for whom immunity wanes. Scenario three is an extension to case two by introducing the terminology of waning immunity.
- *Scenario four*. An additional medical intervention increases immunity duration. We explore immunity duration across a range (10–80 years). This case is a particular variation of case three, where the immunity duration is specified in the defined range. This scenario is to predictively investigate possible future interventions.

## Results

**Figure 3:**
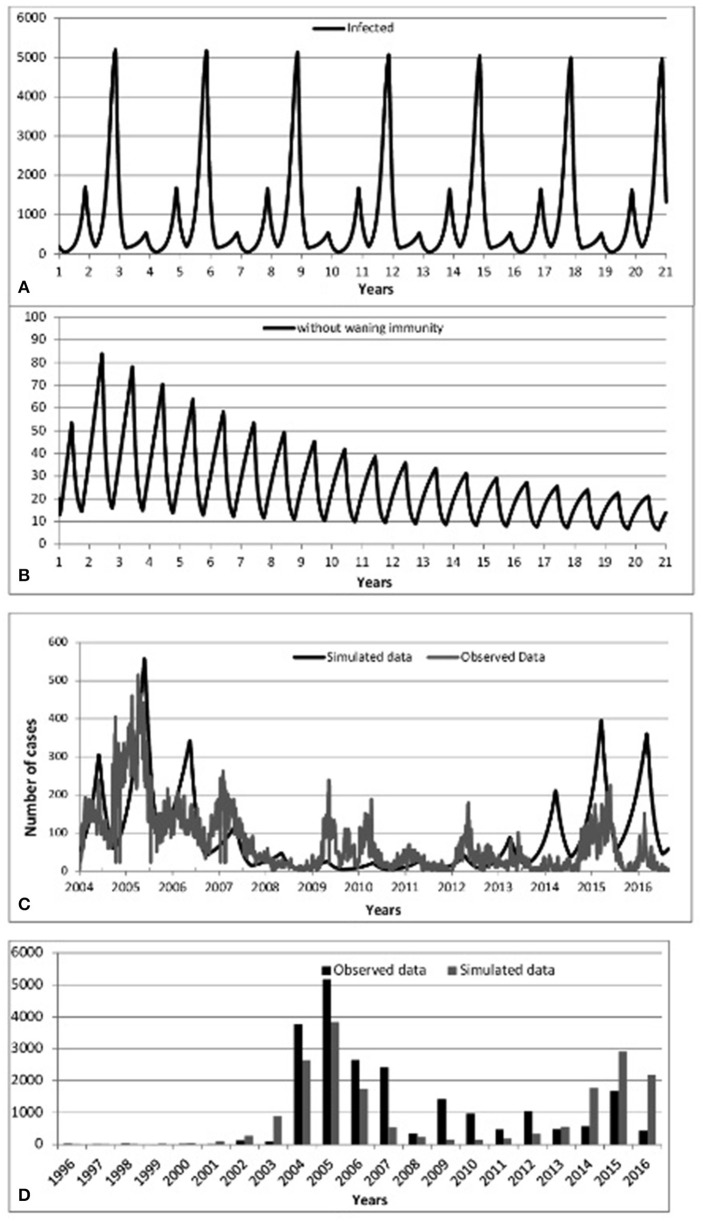
**Predicted incidence of mumps from 2004 to 2016:: (A)** Scenario 1-No vaccination, **(B)** Scenario 2-Vaccination without waning immunity, **(C)** Scenario 3-Vaccination with waning immunity, **(D)** Predicted-Observed data for mumps from 1996 to 2016.

According to observed mumps data in Scotland in Figure 1, and in conjunction with observed mumps data in England and Wales in Figures A1, A2 (see Appendix 3 in Supplementary), three different periods of an epidemiological shift in incidence are observed: pre-vaccine, successful post-vaccine and waning immunity period. Figure 3 depicts time series results for infected cases under scenarios 1–3. Overall, it is clear that mumps occurs every year, regardless of vaccination or waning immunity; however, those factors control the amplitude of the epidemic and the frequency of the highest peaks driving a long term damping oscillation of large outbreaks. After 100 years the difference between the high and low of the cycle is around 25 cases.

*Scenario One (No Vaccination* = *Pre-Vaccine Era)*

We begin by checking model performance without vaccine. Figure 3A shows an inter-epidemic period of 3 years within an oscillatory pattern of mumps cases. This matches parameter values of incubation period of 13 days, infectious period of 7 days and a mean age of infection of 5 years (all within the ranges of Table 1 Appendix 3 in Supplementary). This is supported by the incidence of mumps in England and Wales (Anderson et al., 1987) and observations in the literature reporting cycles of 2–5 years (Galazka et al., 1999).

**Figure 4:**
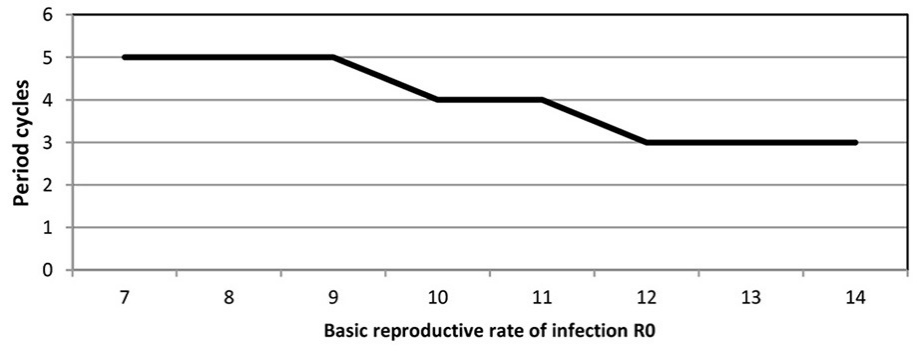
**Inter-epidemic period against basic reproductive rate R0 for pre-vaccine era**.

We point out that predicted cycles do not damp out during 100 years of simulations. By varying seasonality parameter of the model, including removing seasonality altogether, we observed that after a long period the model reaches an endemic state. To further reinforce the suitability of the model we considered R_0_ ranging from [7 to 14]. Figure 4 (see Appendix 3 in Supplementary) shows that increasing R_0_ leads to decreasing the inter-epidemic period from 5 to 3 years.

*Scenario Two (up to two vaccinations and immunity is permanent* = *immediate post-vaccine era)*

Turning to the successful post-vaccine era (and assuming life-long immunity), Figures 3B,D show a massive decrease of mumps infections consistent with observed data 1988–2003, where waning immunity was not yet an important factor and the number of cases overall dramatically decreased due to the decreased pool of susceptibles, in turn due to vaccination. Again, this helps to confirm that the model successfully models historical data.

Scenario Three (up to two vaccinations and immunity wanes)

Figure 3C (resp. Figure 3D) shows model prediction against observations from Scotland in the post-vaccine era (2004–2016, resp. 1996–2016). Figure 3C shows pattern of mumps outbreaks from 2004 to 2016 as waning immunity begins to be more relevant. The simulated data (black solid line) displayed in Figure 3C depicts patterns of mumps dynamics qualitatively similar to observed data (gray solid line). Mumps is notoriously under-reported (Takla et al., 2013) as, especially for those in whom immunity has waned, the disease is often milder (and infected do not seek medical attention). Our model has no notion of “level” of infection, therefore sub-clinical, mild, and serious infections are all counted and contribute to disease transmission. Observed data is scaled by two to compensate for under-reporting of mumps. This is a conservative estimate, based on higher uptake of vaccine in Scotland than in Germany (Takla et al., 2013). This is discussed further in the Discussion.

Figure 3D shows that 2005/2015 years were the dominant period reflecting the highest peaks of mumps infection. Some notable gaps are observed (2009, 2010 and 2012); the observed mumps dynamics are inherently stochastic and noisy. Figures 3C,D depicts that the simulated data for the year 2016 follows the same patterns as observed data, where the number of infected start to decrease. Qualitatively, the simulation results show that even if vaccination is applied, mumps is occurring each year, where the seasonal patterns of our model depict that the infection increases rapidly over the last few months of the year and the high peak is reached early at the start of the year. This is broadly in agreement with observed data.

**Figure 5:**
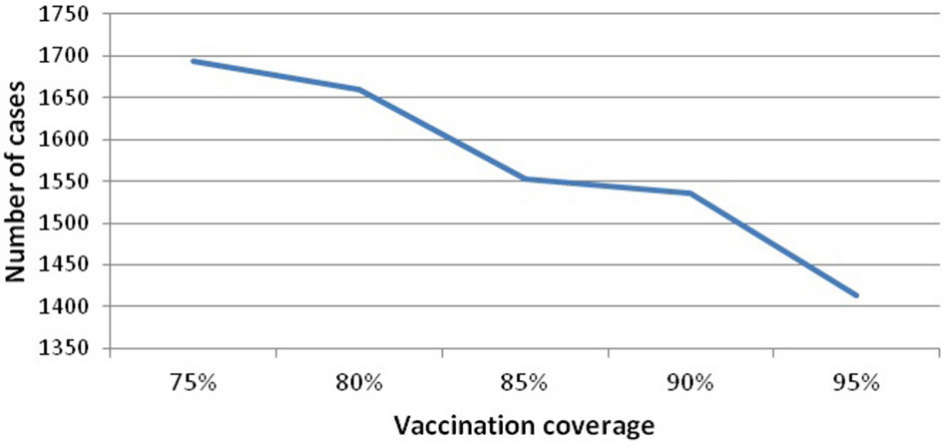
**Infected against vaccination coverage**.

Vaccination coverage dips in this period, but this is not the main factor leading to the resurgence and sustainability of mumps, nor is seasonality on its own (as above). We investigate the variability of vaccination coverage by ranging its value from [75 to 95], where 75% is the minimum value related to the threshold level and 95% is the maximum value of applied vaccine coverage in Scotland. Figure 5 (see Appendix 3 in Supplementary) shows that increasing vaccine coverage leads to a decrease in the peak of infected^7^ (from 1694 to 1413). This is 16%, and still produces a large number of cases. Therefore, increasing the vaccination coverage does not prevent disease occurrence. In addition, we note that all experiments (vaccination coverage ranging from [80 to 95]) settle into a 10 year pattern of gently damping oscillations (100 years of simulation), where the large oscillations are up to 2045, and thereafter the outbreaks become more and more regular in height.

**Figure 6:**
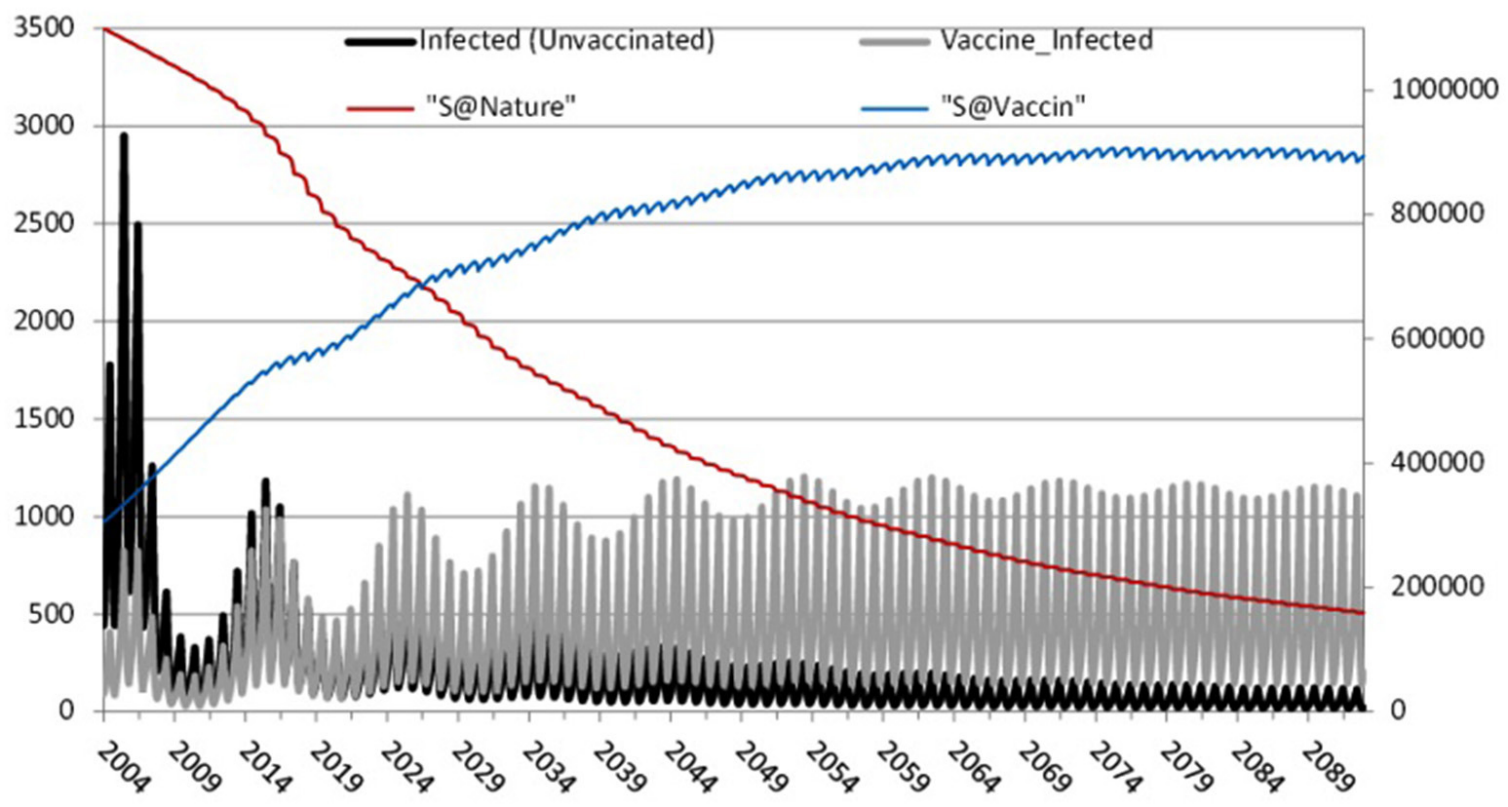
**The effect of waning immunity: Left axis: Infected-unvaccinated, Infected-vaccinated**. Right axis: natively susceptible and modified susceptible.

To further investigate the impact of waning immunity Figure 6 depicts separately those infected-unvaccinated and those infected-vaccinated against natively susceptible and modified susceptible over 100 years of temporal prediction. As expected, due to increasing levels of vaccinated individuals in the population, the number of natively susceptibles and infected-unvaccinated decreases over time, reaching a steady state of infection of around 200 individuals. Conversely, waning immunity leads to an increase in the number of modified susceptible and infected-vaccinated, settling into a 10 year pattern with peaks of between 800 and 1200. Therefore, waning immunity and its effects are the dominant portion of any epidemic.

Scenario Four (additional booster-up to three vaccinations and immunity wanes)

**Figure 7:**
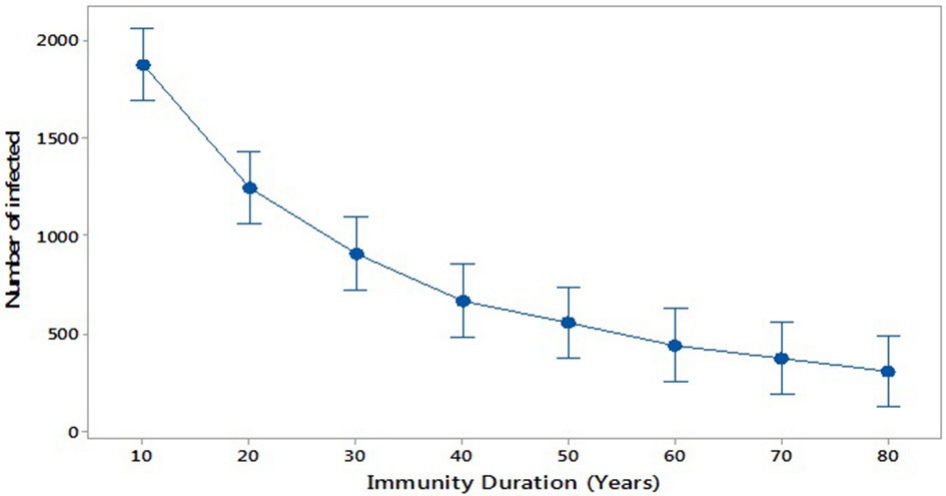
**Infected against duration of immunity**.

Further, we consider scenario 4: the impact of increasing the period of immunity by applying an additional dose of MMR (R and. Cameron, 2016). This could be similarly done by increasing immunity by increasing the efficacy of the vaccination (Public Health England, 2013). We investigate increasing immunity duration in steps from 10 to 80 years (broadly, life expectancy). Figure 7 compares these scenarios and shows that the average of the number of infected individuals at the peak of each outbreak decreases with increasing duration of immunity, as expected.

## Sensitivity analysis

The results above depend on precise parameter values, therefore we used sensitivity analysis to show that the qualitative results of periodic large outbreaks hold across the range. We identify significant parameters reproducing first the observed data, and second leading to the low level endemic state. Table 2 Appendix 3 in Supplementary shows the impact on epidemic amplitude and the periodicity of damping cycles of a series of experiments during 100 years of simulation varying model parameter values for: transmission rates (β1, β2, β3), infectious period (γ), incubation period (α), immunity duration (τ, δ), and vaccination rate (μ1, μ2, μ3). The values of the remaining parameters (birth rate, death rate and immigration rate) are fixed.

**Figure 8:**
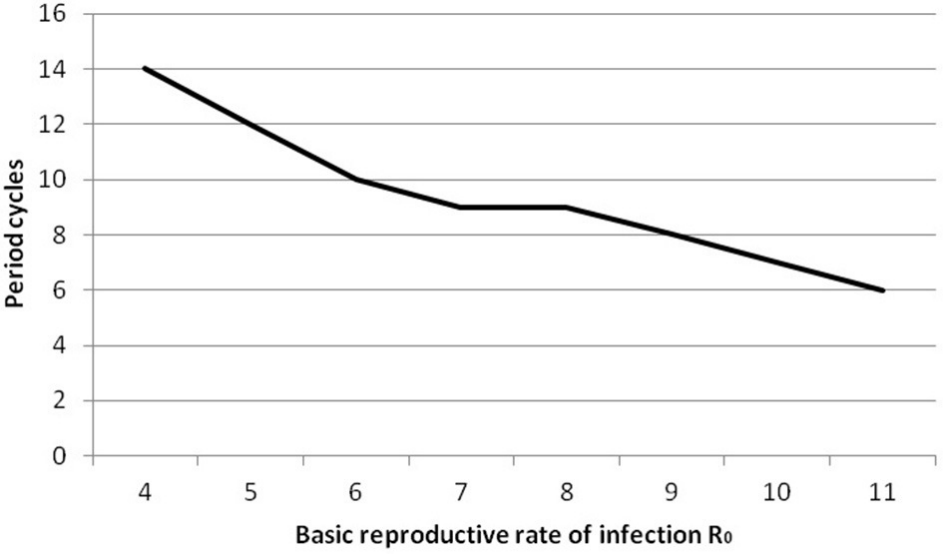
**Inter-epidemic period against basic reproductive rate R0 for post-vaccine era**.

For all analysis we used ANOVA as implemented in Minitab (Minitab 17 Statistical Software, 2010). The full details of the analysis are in Appendix 2 in Supplementary: as expected, only varying transmission rates and immunity duration impact on results. Increasing R_0_ leads to a decrease in period between large outbreaks and therefore an increase in the number of oscillations (see Figure 8, Appendix 3 in Supplementary). Smaller immunity durations increase the pool of susceptibles faster and therefore lead to larger and earlier epidemics.

## Discussion

Our analysis shows that mumps epidemics will continue, with larger outbreaks of ~1200 every 10 years as shown in Figure 6, eventually settling into an endemic state. This is despite high vaccination coverage against mumps (87–95%) since 1988 in Scotland^8^ (well above the estimated herd immunity threshold of 75–86% Donaghy et al., 2006).

In this paper, we have presented the results of mathematical modeling using Bio-PEPA, identifying the impact of vaccination and waning immunity in the mumps component of the MMR vaccine. Even though vaccination has been ongoing since 1988, thus largely preventing mumps in children, our results show that waning immunity is the main factor in a repeated pattern of outbreaks. Simulations and analysis undertaken showed that waning immunity over 10 years leads to the highest number of infected and to the longest inter-epidemic period for larger outbreaks.

The first part of this study was to build a seasonal model which reproduces the patterns of the observed data in three scenarios: no vaccination, initial post-vaccine period with immunity for life, and with waning of vaccine-induced immunity as suggested by several sources (Dayan et al., 2008; Snijders et al., 2012; Abrams et al., 2014; Park, 2015). Those show that mumps is present in previously vaccinated individuals with the majority of those affected being university students. While based on Scottish data this is not a peculiarly Scottish phenomenon: for example, in the US (Harling et al., 2005), Korea (Park, 2015) and the Netherlands (Snijders et al., 2012) adolescent individuals were notified as infected despite high vaccine coverage. In these countries, it was observed that the majority of cases were in young adult (18–25 years) who have been fully vaccinated. In the US, where the first dose of MMR was introduced in 1977 and the second dose in 1990, the outbreak occurring in 2006 reached 6584 cases, 63% of whom received two doses of vaccine. For this country it was reported that in 1982 the incidence rate was reduced to 97% and the 3 year cycles observed in the pre-vaccine era disappeared. Moreover, in 2005, 1 year before the resurgence of the outbreak occurred in 2006, the incidence rate was damped to up to 99% where the vaccine coverage reached 91.5%. In the Netherlands, the large epidemic which occurred in 2004 led to the reintroduction of mumps as a notifiable disease. This followed its removal from the notifiable disease register in 1999 as a consequence of low outbreaks and vaccination coverage of at least one dose of MMR of at least 93% since the introduction of routine vaccine in 1987. In Korea, the epidemic of 2013–2014 showed that 99% of infected individuals aged from 13 to 18 years have been fully vaccinated. It is worth noting that Korea is not that different from other countries as in the pre-vaccine era the epidemic cycles were identified at 4 to 5 years and the mean age of infection at 4 to 6 years which shifted to teenagers in the recent outbreaks (2007 and 2013) in time when vaccination coverage rose to 90%.

Waning immunity is expressed in our model by including an additional compartment of modified susceptible, which is increased by vaccinated individuals (MMR1 and MMR2) losing their immunity. We find that assuming 5 years of MMR1 vaccine-induced immunity (resp. 10 years of MMR2 vaccine-induced immunity) generates simulation results consistent with more recent mumps post-vaccine data from Scotland (2004–2015). In addition, as our model suggests a 10-year-long gradually damping oscillation, the following trajectory of mumps disease would show a decrease in 2016 and so on, building back up from 2020 to another high peak in the year 2025. The most recent data provided by HPS has confirmed this prediction, where the year 2016 depicts 215 cases compared to 2015 which defines 836 cases. Although our estimates of the amplitude of mumps epidemics are higher than observed data, we conjecture that this can be explained by a low level of reporting. Anecdotally, cases of mumps in vaccinated individuals have much milder symptoms and therefore may be undetected (Public Health England, 2013; Takla et al., 2013; Cordeiro et al., 2015; Gouma et al., 2016).

By considering different values of immunity duration (scenario 4) we can estimate the time needed to reverse the epidemic trend and eliminate mumps. This models the situation that, for example, a new, more effective, vaccine is introduced, or a third vaccine dose is introduced into the national programme. This is shown in Figure 7. Even extending immunity to 80 years, a reasonable lifespan, mumps outbreaks still occur. Only by further increasing immunity duration to 150 years eliminates mumps outbreaks, assuming no perturbations occur, such as a new vaccine or new strain of mumps.

It is worth noting that the basic reproductive number R_0_ for the pre-vaccine era is estimated at 10.5 which falls in the range [7–14] as cited in literature (Anderson and May, 1992; Keeling and Rohani, 2008) and for the post-vaccine era R_0_ is estimated at 6 where in the literature it is quoted at [4–7] (Anderson and May, 1982). Recall that R_0_ indicates the number of secondary infections, clearly showing that the number of doses of vaccination and immunity duration has a great impact on decreasing infectious contacts.

Cumulatively, our findings suggest that the more “unprotected” individuals (who were either never vaccinated or lost their immunity), the shorter the period between two high peaks of epidemic outbreak (note the number of cycles in Table 2 for varying values of R_0_). In addition, in both cases related to scenarios 1 and 3 (No vaccination and waning immunity), an earlier high peak of mumps is expected. This occurs because the pool of susceptibles is increasing faster as those vaccinated lose their immunity and move to the susceptible state (scenario 3), or the pool of susceptibles is decreasing faster when no vaccination is applied and R_0_ is higher (scenario 1). Clearly, controlling the number of susceptible individuals has a great impact on controlling disease. As argued by Gay (1998): to achieve elimination of an epidemic, low levels of susceptible individuals should be maintained, leading the basic reproductive number (R_0_) to be <1. We do this here by adjusting immunity duration.

These conclusions illustrate an enhanced understanding of mumps disease in response to mass immunization gained through mathematical modeling. Further, our multi-disciplinary team could explore the potential impact of further vaccination on cyclic outbreaks. Our conclusion for public health services is that they should urge vaccine uptake in those eligible since a high degree of protection is offered by the vaccine overall for those under 18. Considering the possible economic cost/benefit of a third vaccine dose, it seems that while there would be an increased period of immunity, the cyclic outbreaks would continue at about 2/3 the current level, therefore this would not offer significant advantages over the present situation. The Joint Committee on Vaccination and Immunization^9^ do not consider these large outbreaks of particular concern, since there has been no formal discussion to introduce a 3rd vaccine dose into the national programme.

We suggest further study with this model could include vaccination programmes targeted to those subject to waning immunity or at higher risk due to social mixing in a diverse population (as in higher education). Such a model might also include economic factors to allow the effect of targeted programmes to be more precisely evaluated. Another interesting facet would be to bring more attention to the level of immunity by analyzing the vaccine/virus content and detect eventual discrepancy between vaccine strain and mumps outbreak. This might also be linked with a data science approach to analyzing serology of confirmed cases. There are further opportunities to use data science to analyse other features, such as geographic distribution. These developments would allow an enhanced version of Figure 6 showing waves of outbreaks related to waning immunity, evolution of strains of mumps, and locality.

## Author contributions

The Conception or design of the work: CS, DH, and KP. Data collection: RC, KP. Data analysis and interpretation: CS, DH, and KP. Drafting the article: DH, CS. Critical revision of the article: CS, KP, DH, and RC. Final approval of the version to be published: CS, KP, DH, and RC.

## Funding

DH is grateful to the Algerian government, the University of Mostaganem and to the University of Oran for supporting her series of research visits to the University of Stirling.

### Conflict of interest statement

The authors declare that the research was conducted in the absence of any commercial or financial relationships that could be construed as a potential conflict of interest.

## Footnotes

^1^Department of Health Protection Scotland, National surveillance center.

^2^Department of Health Protection Scotland, National surveillance center.

^3^Department of Health Protection Scotland, National surveillance center.

^4^*Information Service Division. Childhood Immunisation Statistics*, Available online at: http://www.isdscotland.org/Health-Topics/Child-Health/Immunisation/

^5^Department of Health Protection Scotland, National surveillance center.

^6^*R. Cameron. Health Protection Scotland, Immunization Team. Personal communication*. (Accessed Jan 21, 2016).

^7^Average number of infected corresponds to the average of the highest peaks during 100 years of simulations.

^8^Department of Health Protection Scotland, National surveillance center.

^9^UK body advising government health policy on vaccination and immunisation.
