# Supplementary material for: Waning Immunity Is Associated with Periodic Large Outbreaks of Mumps: A Mathematical Modeling Study of Scottish Data
Source: Front Physiol. 2017 Apr 25;8:233. doi: 10.3389/fphys.2017.00233 (PMC5404202; doi:10.3389/fphys.2017.00233)
Supplement: Supplementary file 1 [file DataSheet1.docx]

# Appendix 1: Bio-PEPA Model

A Bio-PEPA model, illustrated below, is defined by three main components: species, functions of species dynamics and rates at which those species evolve. Modelling mumps in Bio-PEPA requires describing fully those features accordingly to the model in Fig. 2 and its description above.

*Rates*. All rates fully described in Table 1 are reported in Bio-PEPA code, from line 1to 12. In addition, Bio-PEPA defines the parameter “location” (from line 13 and line 18). As in our model, the population is considered homogeneous, therefore all individuals belong to the same space. Seasonality is expressed by using the Heaviside function (H). As noted by Marco et al. [53]: ” Heaviside function (H) is used to switch customised behaviours on or off in the kinetic laws, this gives a binary valued function from time”. The lines from 19 to 22 code two seasons. The system moves instantaneously from the high epidemic season defined from October to May to the low epidemic season defined from June to September.

*Species and Functional rates (KineticLawOf).* According to the compartments shown in Fig. 2, seven species are defined: S1, S2, V1, V2, E, I, R. Species carry out actions (**kinetic laws)** leading to increase/decrease their level (from line 24 to 40). Actions occur at a rate determined by the kinetic law. Most of these kinetic laws are simple mass action terms defined by the parameters described in the Table 1. Since species interact, the dynamics of each species may affect the level of other species. The scale of this dynamic is bounded by the functional rate specified for each species. For example the action described in line 34, related to incubation and used both by species “E” and “I”, leads to a decrease in the Exposed species (line 43) expressed by the operator “<<”, while it leads Infected species to increase using the operator “>>”. Bio-PEPA species can carry out different activities at each time step, by using the operator ‘+’.

The last line of the model (line 48) defines the interaction between species, and their initial sizes.

**Parameters**

1 D_R = 0.000037;

2 Beta1 =0.80;

3 Beta2 =1.03;

4 Beta = 0.45;

5 Mu2= 0.0000028;

6 Mu3= 0.000025;

7 Mu1 = 0.0000021;

8 Alpha = 0.05;

9 Gama = 0.167;

10 imrate1 =0.07;

11 Tau= 0.00034;

12 Delta=Tau/2;

13 sizeOutside = 110000;

14 sizeLocal = 5300000;

15 location world : size =5200000 , type = compartment;

16 location Local in world: size = sizeLocal, type = compartment;

17 location Local in world: size = sizeLocal, type = compartment;

18 location Outside in world : size = sizeOutside, type = compartment;

19 thigh = 4;

20 tlow = 9;

21 month = floor(time/30);

22 season_time = 1-H( ((month - 12*floor(month/12)) - tlow)*(thigh-(month - 12*floor(month/12))) );

23 N = (S1@Local +E@Local + I@Local + R@Local +S2@Local + MMR1@Local + MMR2@Local);

**Kinetic Laws**

24 kineticLawOf BIRTH1: Mu1 * N;

25 kineticLawOf BIRTH2: Mu2 * N;

26 kineticLawOf BIRTH3: Mu3 * N;

27 kineticLawOf MMR1_S2: MMR1@Local *Tau;

28 kineticLawOf MMR2_S2: MMR2@Local *Delta;

29 kineticLawOf Death_MMR1 : D_R * MMR1@Local;

30 kineticLawOf Death_MMR2 : D_R * MMR2@Local;

31 kineticLawOf immigration : imrate1/10000;

32 kineticLawOf S1_E: (Beta1 * S1@Local * I@Local)/N * (season_time)

+ (1-season_time)*(Beta * S1@Local * I@Local)/N ;

33 kineticLawOf S2_E: (Beta2 * S2@Local * I@Local)/N * (season_time)

+ (1-season_time)* (Beta * S2@Local * I@Local)/N;

34 kineticLawOf E_I: Alpha * E@Local;

35 kineticLawOf I_R: Gama * I@Local;

36 kineticLawOf Death_S1: D_R * S1@Local;

37 kineticLawOf Death_I: D_R * I@Local ;

38 kineticLawOf Death_E: D_R * E@Local;

39 kineticLawOf Death_S2: D_R * S2@Local;

40 kineticLawOf Death_R: D_R * R@Local;

**Species**

41 S1 = (BIRTH1,1) >> S1@Local + (S1_E,1) << S1@Local + Death_S1 << S1@Local;

42 S2 = (S2_E,1) << S2@Local + Death_S2 << S2@Local + (MMR2_S2,1) >> S2@Local +(MMR1_S2,1) >> S2@Local;

43 E = (S1_E,1) >> E@Local +(S2_E,1) >> E@Local +(E_I,1) << E@Local+ Death_E << E@Local;

44 I = (E_I,1) >> I@Local +(I_R,1) << I@Local + Death_I << I@Local + immigration[Outside -> Local](.)I

+ (S1_E,1) (.) I+ (S2_E,1) (.) I;

45 R = (I_R,1) >> R@Local+ Death_R << R@Local ;

46 MMR1 = (BIRTH2,1) >> MMR1@Local + (MMR1_S2,1) << MMR1@Local+ Death_MMR1 << ;

47 MMR2 = (BIRTH3,1)>> MMR2@Local + (MMR2_S2,1) << MMR2@Local + Death_MMR2 << ;

**Model component**

48 S1@Local[1100000]<*> S2@Local[305500]<*> E@Local[0]<*> I@Local[20]<*> R@Local[3018600]<*> MMR1@Local[29250] <*>MMR2@Local[276250] <*> I@Outside[100000]

# Appendix 2: Sensitivity analysis

1: Incubation period experiments

The analysis per ANOVA is carried out for 14 experiments where the incubation period varied from 12 to 25 days per one step day. The results indicate that at 95% of confidence, no significant statistical differences between experiments (p = 0.968) and then the null hypothesis (the means of experiments are equal) cannot be rejected. Hsu’s MCB test and Tukey test imply that varying incubation period does not affect the number of infected; however, using simulations we can look at cycles. 100 years of simulations show that by increasing the incubation period the periodicity changes from 8 to 11 cycles.

Analysis 2: Infectious period experiments

Varying the infectious period from 6 to 9 days per one step day, indicates no significant statistical differences ( p= 0.114). However, the results validated by the Tukey test are in contrast with the Hsu’s MCB test results. While the former shows no significant differences, the latter shows significant differences between an infectious period of 6 days (1^st^ experiment) and the one of 9 days (4^th^ experiment). In fact, the analysis shows clearly that the mean of the 4^th^ experiment (2739) is higher than the others (1808, 2113, 2276). In addition the simulation results show that increasing the infectious period increases the amplitude of the epidemic where the main gap is depicted at the first peak.

Analysis 3: Transmission rates experiments

Transmission rate experiments are based on changing the basic reproductive number R_0_ from 4 to 11. This equates to varying the high transmission rate from 0.44 to 1.83 and the low transmission rate from 0.19 to 0.81. ANOVA analysis shows that experiments are not statistically significantly different (p = 0.36). However, simulations over 100 years indicate that increasing the basic reproductive number leads to a decrease in periodicity. As R_0_ varies from 4 to 11 the period of cycles per 100 years of simulation varies from 14 to 6 and the number of cycles varies from 7 to 16 cycles. During simulations, it was observed that the first epidemic tends to occur sooner with increasing amplitude as R_0_ increases.

Analysis 4: Immunity duration experiments

The analysis per ANOVA of the different values of immunity duration varying from 10 to 80 years, reveals statistically significant differences. In particular, the analysis depicts four different groups. The first group includes only one experiment (immunity duration = 10 years). The second group includes two experiments (immunity duration = 20 and 30). The third group includes three experiments (30, 40 and 50). The fourth group includes five experiments (40, 50, 60, 70 and 80), where the 2^nd^ group overlaps the third group with one experiment (30) and the third group overlaps the fourth group with two experiments (40, 50). In ANOVA, the experiment which does not share any group is considered significantly different. This implies that experiment one (10) is significantly different from all others. This is because small immunity duration tends to increase the pool of susceptibles faster and the epidemics occur sooner with higher amplitude. Moreover, this analysis supports the idea that immunity duration has a major effect on the epidemic dynamics, while varying incubation period, infectious period and transmission rates do not show such large impact on epidemic curves.

Analysis 4: Vaccination coverage experiments

Varying vaccination coverage from 75% to 95% in steps of 5 percentage points, indicates at 95% of confidence no significant statistical differences (p= 0.648) between experiment and H0. The results validated by Tukey test are similar to those with Hsu’s MCB test results which imply that varying vaccination coverage does not affect the number of infected; this fact is confirmed by simulations performed where we can look at cycles. 100 years of simulations show that by increasing the vaccination coverage the periodicity does not change significantly. From 80% to 95% the simulations detect 10 cycles where at 75%, the periodicity of cycles is at 9 years. These findings support the conclusions of DeStefano et al [15] and Donaghy et al [14].

# Appendix 3: Mumps data in England and Wales

**Table 1.** Model parameters

| Parameter | Description | Value (day) | Formula |
| --- | --- | --- | --- |
| B | Birth rate | 3 10^-5^ | Number of birth / Total population |
| μ | Death rate | 3.7 10^-5^ | Number of death / Total population |
| μ_1_ | No-vaccination rate | 2.1 10^-6^ | Birth rate -(μ_2_+μ_3_) |
| μ_2_ | Vaccination rate (MMR1) | 2.8 10^-6^ | Birth rate * VC1 |
| μ_3_ | Vaccination rate (MMR2) | 2.5 10^-5^ | Birth rate * VC2 |
| τ | Waning immunity rate (MMR1) | 3.4 10^-4^ | 1/immunity duration of MMR1 |
| δ | Waning immunity rate (MMR2) | τ/2 | 1/immunity duration of MMR2 |
| β1  β2  β3 | Transmission rate for :  - high season and native susceptible  - high season and modified susceptible  - low season | 0.7  0.9  0.4 | β = R_0_ * γ |
| T^[[1]](#footnote-1)^ | Inter-epidemic period | [2-5] | T = 2π * $\sqrt{A(\frac{1}{\alpha}+\frac{1}{})}$ [42]  where A: mean age of infection |
| 1/α | Incubation period | [12-25] | 1/infection rate |
| 1/γ | Infectious period | [7-9] | 1/recovery rate |
| λ | Immigration rate | 0.07 | Immigration ∗$\sqrt{\mathbf{population}}$ |

**Table 2.** Sensitivity analysis summary

| **Incubation period** | | |  | |  | | |  | | | | |  | | |  | | | |  | | |  | | | |  | |  |  |  | |  | |  | |  |
| --- | --- | --- | --- | --- | --- | --- | --- | --- | --- | --- | --- | --- | --- | --- | --- | --- | --- | --- | --- | --- | --- | --- | --- | --- | --- | --- | --- | --- | --- | --- | --- | --- | --- | --- | --- | --- | --- |
| Values | | | 12 | | 13 | | | 14 | | | | | 15 | | | 16 | | | | 17 | | | 18 | | | | 19 | | 20 | 21 | 22 | | 23 | | 24 | | 25 |
| Amplitude | | | 2357 | | 2316 | | | 2229 | | | | | 2123 | | | 2020 | | | | 2309 | | | 2280 | | | | 2153 | | 2149 | 2132 | 2020 | | 1968 | | 1909 | | 1927 |
| Period of Cycles | | | 8 | | 9 | | | 9 | | | | | 9 | | | 9 | | | | 9 | | | 10 | | | | 10 | | 10 | 10 | 11 | | 10 | | 11 | | 11 |
| **Infectious period** | | |  |  | | | | | |  | | | | |  | | | | |  |  |  |  |  |  |  |  |  |  |  |  |  |  |  |  |  |  |
| Values | | | 6 | 7 | | | | | | 8 | | | | | 9 | | | | |  |  |  |  |  |  |  |  |  |  |  |  |  |  |  |  |  |  |
| Amplitude | | | 1808 | 2132 | | | | | | 2276 | | | | | 2739 | | | | |  |  |  |  |  |  |  |  |  |  |  |  |  |  |  |  |  |  |
| Period of Cycles | | | 10 | 10 | | | | | | 11 | | | | | 10 | | | | |  |  |  |  |  |  |  |  |  |  |  |  |  |  |  |  |  |  |
| **Basic reproductive number** | | | | | | | | | | | | | | |  | | | |  | | | |  | | | |  |  | |  | | |  |  |  |  |  |
| Values | | | | 4 | | | | | 5 | | | | | | 6 | | | | 7 | | | | 8 | | | | 9 | 10 | | 11 | | |  |  |  |  |  |
| Amplitude | | | | 1690 | | | | | 1708 | | | | | | 2132 | | | | 2134 | | | | 2256 | | | | 2320 | 2289 | | 2407 | | |  |  |  |  |  |
| Period of Cycles | | | | 14 | | | | | 12 | | | | | | 10 | | | | 9 | | | | 9 | | | | 8 | 7 | | 6 | | |  |  |  |  |  |
| **Immunity duration** | | | | | | | | | | | |  | | | | |  | | | | |  | | | |  | |  | |  | |  | |  |  |  |  |
| Values | | | | | | | 10 | | | | | 20 | | | | | 30 | | | | | 40 | | | | 50 | | 60 | | 70 | | 80 | |  |  |  |  |
| Amplitude | | | | | | | 1873 | | | | | 1245 | | | | | 909 | | | | | 668 | | | | 555 | | 440 | | 371 | | 306 | |  |  |  |  |
| Period of Cycles | | | | | | | 10 | | | | | 8 | | | | | 7 | | | | | 7 | | | | 6 | | 5 | | 5 | | 4.5 | |  |  |  |  |
| **Vaccination coverage** | | | | | | | | | | | | | | | | | | | | |  | | | |  | | |  |  |  |  |  |  |  |  |  |  |
| Values | | | | | | 75 | | | | | 80 | | | | | | 85 | | | | 90 | | | | 95 | | |  |  |  |  |  |  |  |  |  |  |
| Amplitude (100 years peaks) | | | | | | 1694 | | | | | 1660 | | | | | | 1552 | | | | 1536 | | | | 1413 | | |  |  |  |  |  |  |  |  |  |  |
| Amplitude (10 first peaks) | | | | | | 1602 | | | | | 1587 | | | | | | 1547 | | | | 1504 | | | | 1410 | | |  |  |  |  |  |  |  |  |  |  |
| Period of Cycles | | | | | | 9 | | | | | 10 | | | | | | 10 | | | | 10 | | | | 10 | | |  |  |  |  |  |  |  |  |  |  |


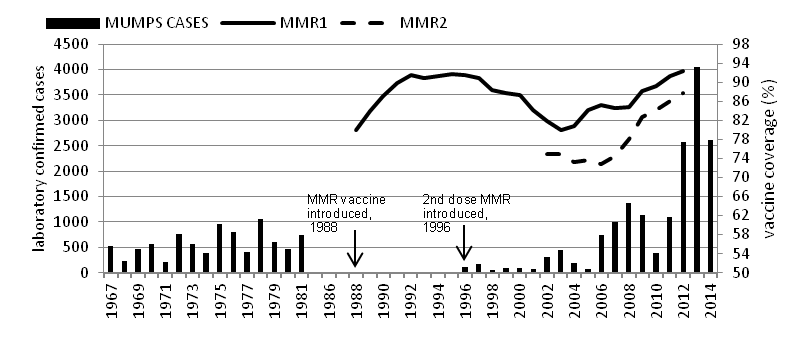


**Figure A1.** Confirmed mumps cases, England and Wales and MMR vaccine coverage: Data from 1967 to 2014 excluding (1982-1995) (Galbraith et al., 1984).


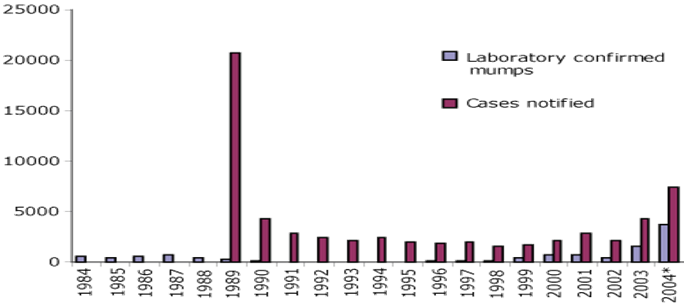


**Figure A2.** Confirmed mumps cases, England and Wales: Data from 1984 to 2004 (Savage et al., 2004).

1. Inter-epidemic period related to a pre-vaccine era [↑](#footnote-ref-1)
